# Supplementary material for: Long term survival and abnormal liver fat accumulation in mice with specific thymidine kinase 2 deficiency in liver tissue
Source: PLoS One. 2023 Oct 5;18(10):e0285242. doi: 10.1371/journal.pone.0285242 (PMC10553353; doi:10.1371/journal.pone.0285242)
Supplement: S1 Table — (DOCX) [file pone.0285242.s001.docx]

| Gene | Forward primer | Reverse primer |
| --- | --- | --- |
| *Tk2* | CGACATGCCTGGAGTTCTTC | TGGTACATGAGGCTCAGAGG |
| *dCK* | AAGACTGGCACGACTGGATG | AAGCATTTCTCGGGAGTAGC |
| *Cpt1a* | ACAGTGGGACATTCCAGGAG | AGGAATGCAGGTCCACATCA |
| *Fasn* | TTAGAGCAGGACAAGCCCAA | TCGGGTGAGGACGTTTACAA |
| *Srebp1* | GAGATTTGCGAACTGGACAC | CGGGAAGTCACTGTCTTGGGT |
| *Tfam* | GATGATTCGGCTCAGGGAAA | TGTCTCCGGATCGTTTCACA |
| *Ppargc1a* | GAGAGGCAGAAGCAGAAAG | CTCAATTCTGTCCGCGTTGT |
| *Rmr1* | GCTATGTGGATCAAGGTGGAAAC | AGTGCCAAGGCTCCCGGTAA |
| *Rmr2* | GCTTATTAGCAGAGACGAGGGTTT | TCCCGCTGGTTTGTGTACCA |
| *Rmr2b* | GACGAACCGTCGGGAACT | TGGGCGACCCGGAAA |
| *Gapdh* | CCATCACCATCTTCCAGGAG | GTGGTTCACCCATCACAA |

**Supplementary table 1. Primer sequences for qPCR analysis**
