# Supplementary material for: Long term survival and abnormal liver fat accumulation in mice with specific thymidine kinase 2 deficiency in liver tissue
Source: PLoS One. 2023 Oct 5;18(10):e0285242. doi: 10.1371/journal.pone.0285242 (PMC10553353; doi:10.1371/journal.pone.0285242)
Supplement: S1 Raw images — (PDF) [file pone.0285242.s002.pdf]

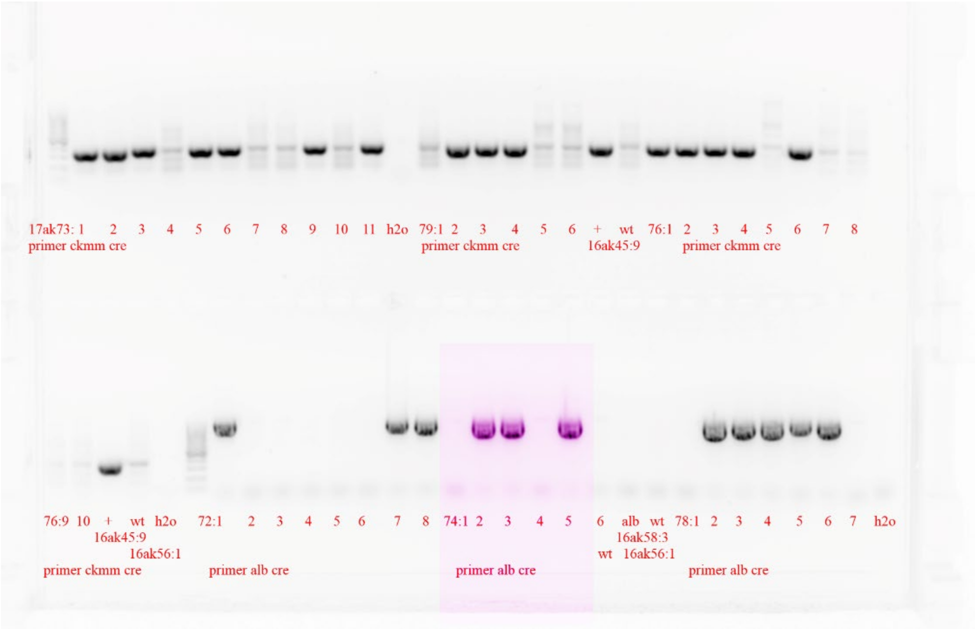

Uncropped genotyping agarose gel result with alb-cre specific F1 and R1 primers. The area with purple background corresponds to Fig1C.

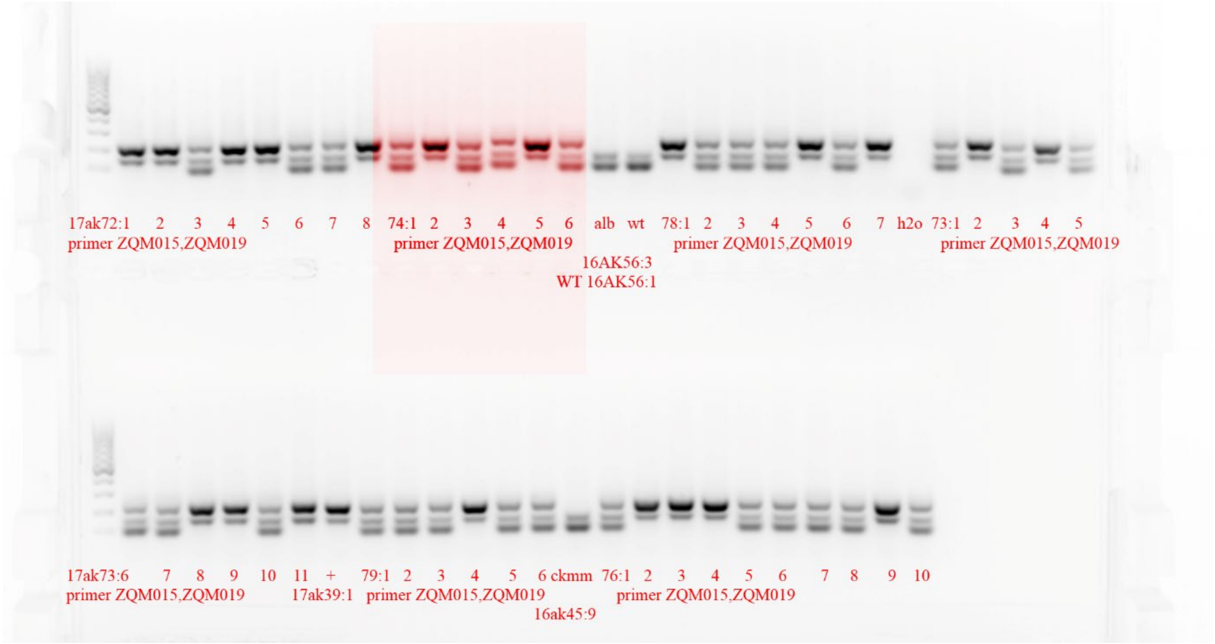

Uncropped genotyping agarose gel result with allele specific F2 and R2 primers. The area with red background corresponds to Fig1D.

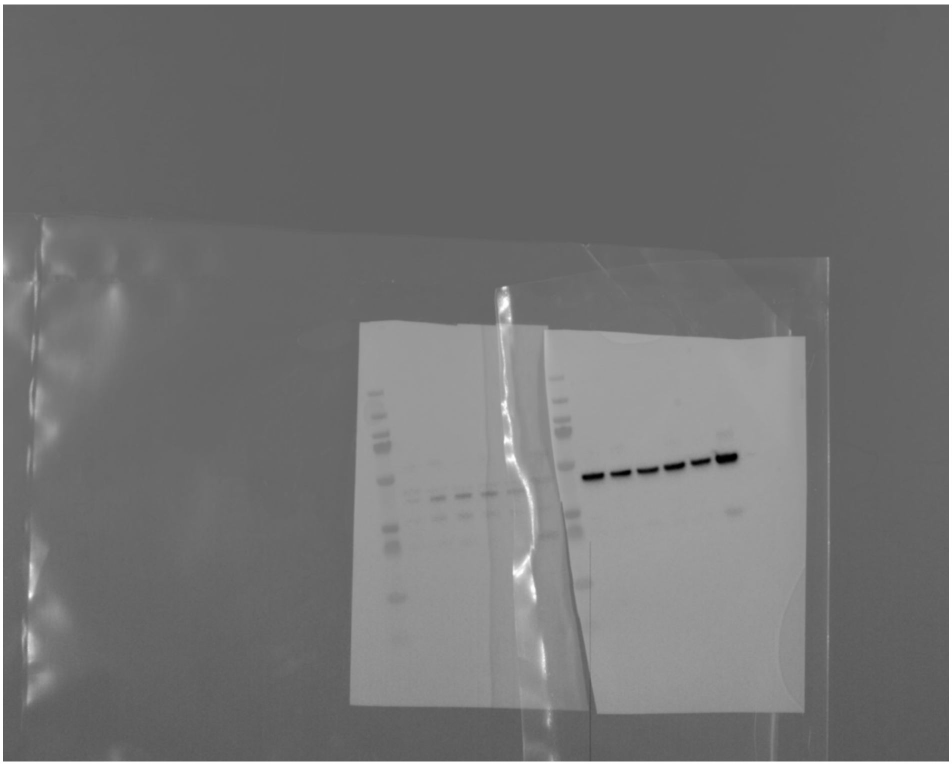

Uncropped western blot with beta-actin primary antibody. The image corresponds to Fig3I.

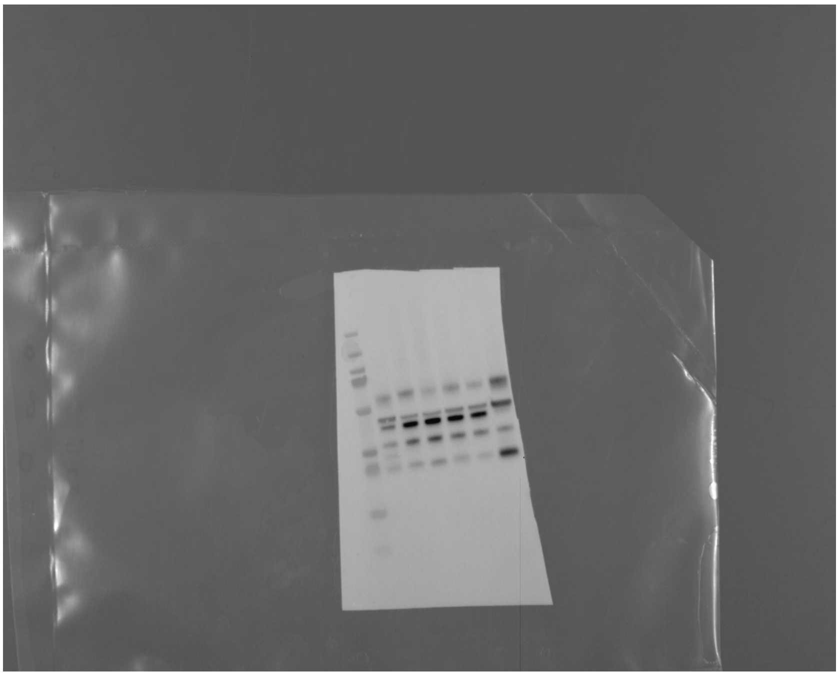

Uncropped western blot with COX1 primary antibody. The image corresponds to Fig3I.

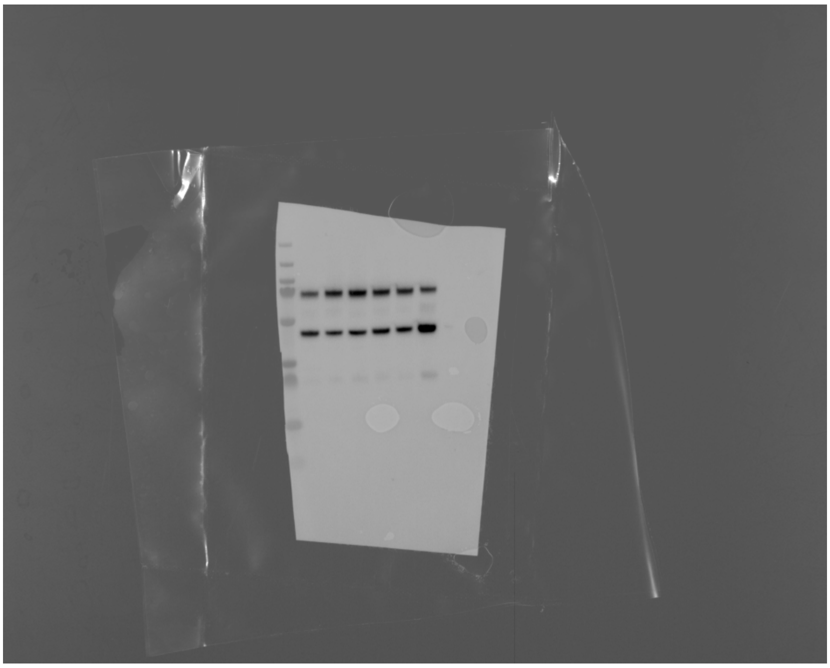

Uncropped western blot with SDHA primary antibody. The image corresponds to Fig3I.
